# Genome-wide identification and expression analysis of proline synthesis and catabolism genes in kiwifruit: exploring the role of *AcP5CS1* in salt tolerance

**DOI:** 10.3389/fpls.2025.1590484

**Published:** 2025-09-03

**Authors:** Jun Yang, Cheng Qian, Can Chen, Guoqi Liu, Xiaowei Zhen, Quaid Hussain, Shiheng Lyu, Chengcheng Ling

**Affiliations:** ^1^ College of Food and Bioengineering, Bengbu University, Beng bu, Anhui, China; ^2^ College of Life Science and Oceanography, Shenzhen University, Shenzhen, China; ^3^ State Key Laboratory of Subtropical Silviculture, Zhejiang A&F University, Hangzhou, China

**Keywords:** kiwifruit, proline metabolism, *AcP5CS1*, transcriptional regulation, abiotic stress

## Abstract

The regulation of proline metabolism is critical for enhancing plant stress tolerance by promoting proline accumulation under abiotic stress conditions. Key enzymes in this pathway include Δ1-pyrroline-5-carboxylate synthase (P5CS), pyrroline-5-carboxylate reductase (P5CR), ornithine δ-aminotransferase (δ-OAT), proline dehydrogenase (PDH), and pyrroline-5-carboxylate dehydrogenase (P5CDH). Despite their importance, comprehensive identification and characterization of these gene families in kiwifruit (*Actinidia chinensis*) remain unexplored. This study identified two *AcP5CSs*, one *AcP5CR*, one *AcOAT*, three *AcPDHs*, and one *AcP5CDH* within the kiwifruit genome. This research comprehensively examined phylogenetic tree, gene structure, motif analysis, cis-regulatory elements and chromosomal distributions analysis, as well as expression profiles under abiotic stresses and hormonal stress. Under salt stress, transcriptional profiling showed marked upregulation of *AcP5CS1, AcP5CR*, and *AcOAT*, while *AcP5CDH* was significantly suppressed, as confirmed by qRT-PCR. Functional analysis demonstrated that *AcP5CS1* overexpression in Arabidopsis significantly enhanced salt tolerance. The correlation results indicated a strong association between the *AcNAC30* transcription factors (TFs) and the expression of *AcP5CS1*. Mechanistic studies using dual-luciferase and electrophoretic mobility shift assays (EMSA) confirmed that *AcNAC30* directly binds to the *AcP5CS1* promoter. Therefore, we speculated that *AcNAC30* likely enhances proline accumulation under salt stress by upregulating the expression of proline metabolic pathway genes. These findings elucidate the genomic architecture of proline metabolic genes in kiwifruit and establish their pivotal role in mediating abiotic stress tolerance.

## 1 Introduction

Plants are constantly exposed to various environmental stresses, including abiotic stresses such as drought, extreme temperatures, salinity, and heavy metal toxicity (Zhu, 2016). These stressors significantly impact plant growth and development, reducing yield and economic losses (Zhang et al., 2022). Given the rising frequency and severity of abiotic stresses caused by climate change, it is essential to comprehend the cellular, physiological, and molecular mechanisms that underlie the plant’s response to these stressors to develop stress-tolerant crop varieties (Peck and Mittler, 2020).

Throughout evolution, plants have developed numerous mechanisms to resist or partially resist external adversity. Osmolytes, which include compatible solutes such as inorganic ions, soluble sugars, glycine betaine, and proline, can rapidly accumulate in response to abiotic stresses and offer beneficial effects in alleviating stress-induced injuries (Ghosh et al., 2021). Proline plays a crucial role in maintaining cellular homeostasis, ensuring turgidity for cell growth and development under abiotic stress conditions (Ashraf and Foolad, 2007; Ghosh et al., 2022; Hosseinifard et al., 2022). Proline accumulation in plants is achieved by coordinating proline biosynthesis, degradation, and transport. Proline biosynthesis in plants involves a series of enzymatic reactions that convert glutamate, an amino acid, into proline (Chun et al., 2018). This process occurs in the cytoplasm and chloroplasts of plant cells and is crucial for the plant’s ability to cope with various environmental stresses (Zhang et al., 2020). Glutamate, an amino acid, serves as the precursor for proline biosynthesis. In the first step of proline biosynthesis, glutamate is converted to γ-glutamyl phosphate by the enzyme Δ1-pyrroline-carboxylate synthetase (P5CS) (Alvarez et al., 2022). This step is considered the rate-limiting step in proline biosynthesis and is highly regulated in response to stress and developmental cues (Qamar et al., 2015). γ-Glutamyl phosphate is first converted to glutamate-5-semialdehyde (GSA) by the enzyme P5CS (Δ1-pyrroline-5-carboxylate synthase). GSA then spontaneously cyclizes to form Δ1-pyrroline-5-carboxylate (P5C). Finally, P5C is reduced to proline by the enzyme P5CR (Δ1-pyrroline-5-carboxylate reductase) (Amini et al., 2015). Additionally, In the ornithine pathway, the amino acid ornithine can be transaminated to form glutamate semialdehyde (GSA) by the enzyme ornithine aminotransferase (OAT) (Yan et al., 2024; Zhang et al., 2024). GSA can subsequently be converted to proline through the activity of the enzyme pyrroline-5-carboxylate synthetase (P5CS) (Geng et al., 2021). The catabolism of proline is facilitated by two rate-limiting enzymes, namely proline dehydrogenase (PDH or ProDH) and Pyrroline-5-carboxylate dehydrogenase (P5CDH) in mitochondria (Sharma and Verslues, 2010). The proline synthesis gene *P5CR1* expression in kiwifruit was upregulated, while, conversely, the proline degradation gene *PDH* expression was downregulated. This regulatory response led to proline accumulation during drought stress (Xia et al., 2022). Overexpressing the *LrP5CS1*、*LrP5CS2*, and *LrP5CS3* genes from *Lilium regale* enhanced osmotic, drought, and salt tolerance in transgenic Arabidopsis without any adverse effects in normal conditions (Wei et al., 2016). The upregulation of *P5CS* gene expression significantly enhances proline accumulation, thereby improving the ability of plants to tolerate stress (Anton et al., 2020). Virus-induced gene silencing further demonstrated the critical role of *GhP5CS1* in cotton’s salt stress response, as silenced plants exhibited heightened sensitivity to salt stress (Fang et al., 2025). After treatment with saline-alkali, the expression levels of *MsP5CSs*, *MsP5CRs*, *MsOATs*, and *MsProTs* from *Medicago sativa L* were significantly upregulated, whereas the expression levels of *MsPDH1.1*, *MsPDH1.3*, and *MsP5CDH* were significantly downregulated (Min et al., 2023). The reduced expression of *KvPDH*, responsible for proline catabolism in *Kosteletzkya virginica*, contributed to proline accumulation under salt stress conditions (Wang et al., 2015). *P5CR* mediates the final step of proline biosynthesis in plants, and the *VvP5CR* gene in grapevines is crucial for drought tolerance (Chen et al., 2021). In addition, the expression of the sugarcane *SsOAT* gene was significantly upregulated in response to drought stress (Yao et al., 2020). Salt stress strongly increases OAT activity in the ornithine pathway, with *OAT* gene expression significantly higher than that of *P5CS1* and *P5CS2* in the glutamate pathway (Guo et al., 2022).

Actinidia, commonly known as kiwifruit, belongs to the climbing or scrambling genus Actinidia and is usually a deciduous perennial vine (Huang et al., 2013). Kiwifruit has become one of the most popular fruits worldwide due to its distinctive flavor and high content of vitamins, minerals, amino acids, and other health-beneficial metabolites (Xiong et al., 2020; Yang et al., 2024; Zeng et al., 2025). However, the vulnerability of the vine to abiotic stresses, including drought, salinity, and low or high temperatures, leads to significant reductions in growth and flowering during the subsequent season, ultimately resulting in diminished fruit quality, low yields, and potentially plant mortality (Hussain et al., 2016). Investigating the genes from kiwifruit that are involved in proline metabolism and directly contribute to proline accumulation during abiotic stresses is essential. Currently, no comprehensive study investigates the evolutionary aspects and expression patterns of the proline metabolism gene family in kiwifruit. Here, we identified candidate genes involved in proline metabolism and investigated their expression patterns in response to abiotic stress conditions. In *A. chinensis*, eight genes, namely, *AcP5CS1, AcP5CS2, AcP5CR, AcOAT, AcPDH1, AcPDH2, AcPDH3*, and *AcP5CDH* involved in proline metabolism pathways were identified. The overexpression of *AcP5CS1* in transgenic Arabidopsis results in enhancing salt tolerance. This indicates that *AcP5CS1* is crucial in improving resilience to salt stress. The molecular mechanisms involved in proline metabolism pathways in kiwifruit have been elucidated through our research, specifically regarding their response to abiotic stresses. These insights serve as a fundamental basis for future molecular breeding strategies to enhance kiwifruit’s stress tolerance.

## 2 Results

### 2.1 Identification and characterization of gene family encoding proline metabolism in the *A. Chinensis* genome

Through a comprehensive exploration of the *A. chinensis* genome, we identified and characterized eight pivotal genes involved in proline metabolism, showcasing an intriguing similarity to the gene family found in Arabidopsis. In Arabidopsis, proline biosynthesis and catabolism are primarily regulated by seven genes: *AtP5CS1* (AT2G39800), *AtP5CS2* (AT3G55610), *AtP5CR* (AT5G14800), *AtOAT* (AT5G46180), *AtPDH1* (AT3G30775), *AtPDH2* (AT5G38710), and *AtP5CDH* (AT5G62530). In *A. chinensis*, our study revealed a corresponding set of eight genes—*AcP5CS1, AcP5CS2, AcP5CR, AcOAT, AcPDH1, AcPDH2, AcPDH3*, and *AcP5CDH*—that displayed notable homology to these Arabidopsis genes (**Table 1**). A comprehensive characterization revealed distinct properties associated with these genes. The protein’ theoretical isoelectric points (pI) diverged substantially, ranging from 5.72 (*AcP5CDH*) to 10.07 (AcP5CR). The translated amino acid (AA) sequences ranged from 236 aa (AcPDH1) to 716 aa (AcP5CS1). Similarly, the molecular weights (MW) of the coded proteins displayed a considerable range from 25.79 kDa (AcPDH1) to 77.33 kDa (AcP5CS2).

**Table 1:** The characterization details of the proline metabolism genes in the kiwifruit genome.

Genome ID	Gene name	Chr ID	Start (bp)	End (bp)	Protein length (aa)	Molecular weight (kDa)	PI
Actinidia39586	*AcOAT*	Chr17	16963627	16977975	474	52.14 kDa	7.65
Actinidia37649	*AcP5CDH*	Chr13	7871589	7880092	436	49.13 kDa	7.36
Actinidia39173	*AcP5CR*	Chr18	11878808	11888616	279	29.00 kDa	10.07
Actinidia13892	*AcP5CS1*	Chr21	14862676	14878328	716	77.33 kDa	6.63
Actinidia05329	*AcP5CS2*	Chr20	10386513	10395602	714	76.87 kDa	5.72
Actinidia17623	*AcPDH1*	Chr21	4139220	4143297	236	25.79 kDa	8.11
Actinidia18108	*AcPDH2*	Chr11	15513606	15516722	463	51.10 kDa	8.29
Actinidia36293	*AcPDH3*	Chr4	3620355	3623539	405	45.06 kDa	8.31

### 2.2 Phylogenetic, structure, motif, cis-element and chromosomal distributions analysis of proline-metabolism gene family

**Figure 1:**
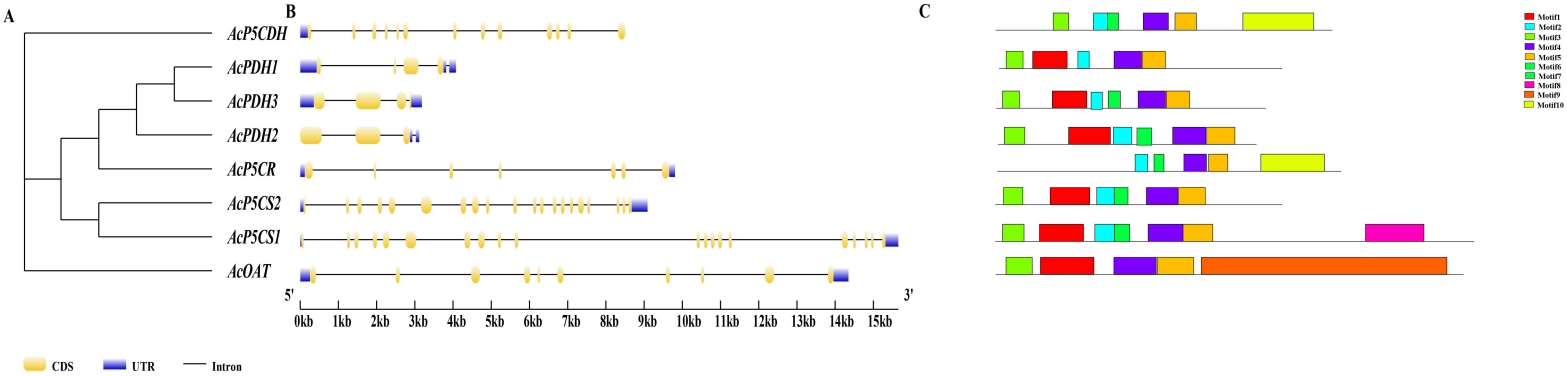
Comprehensive analysis of evolutionary relationship and gene structure in the proline-metabolism gene family. The number on the branch denotes the reliability of the node based on 1000 bootstrap verification. **(A)** The phylogenetic relationships of proline-metabolism gene members were elucidated through a neighbor-joining phylogenetic tree. **(B)** The structural organization of the proline-metabolism gene family gene members is depicted, highlighting the CDS (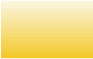), upstream/downstream (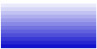), and intron (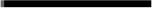). **(C)** The conserved motifs of the proline-metabolism gene family gene members.

In the evolutionary tree, *AcPDH1*, *AcPDH2*, and *AcPDH3* are clustered together, with *AcP5CS1* and *AcP5CS2* demonstrating the closest relationship (**Figure 1A**). Gene structure analysis revealed that *AcP5CS1* and *AcP5CS2* each contain 20 exons and 20 introns, indicating a complex genomic architecture. In contrast, the exon counts for the remaining four genes are as follows: *AcP5CR* has seven exons, *AcOAT* has 10 exons, *AcPDH1* contains four exons, *AcPDH2* contains three exons, *AcPDH3* contains three exons, and *AcP5CDH* has 13 exons (**Figure 1A**). Additionally, all six genes are characterized by upstream and downstream non-coding regions, which play crucial roles in transcriptional regulation (**Figure 1B**). The same conserved motifs in homologous *AcPDHs* and *AcP5CSs* genes might have similar functions and correlations between evolutionary relationships and conserved motifs (**Figure 1C**).

**Figure 2:**
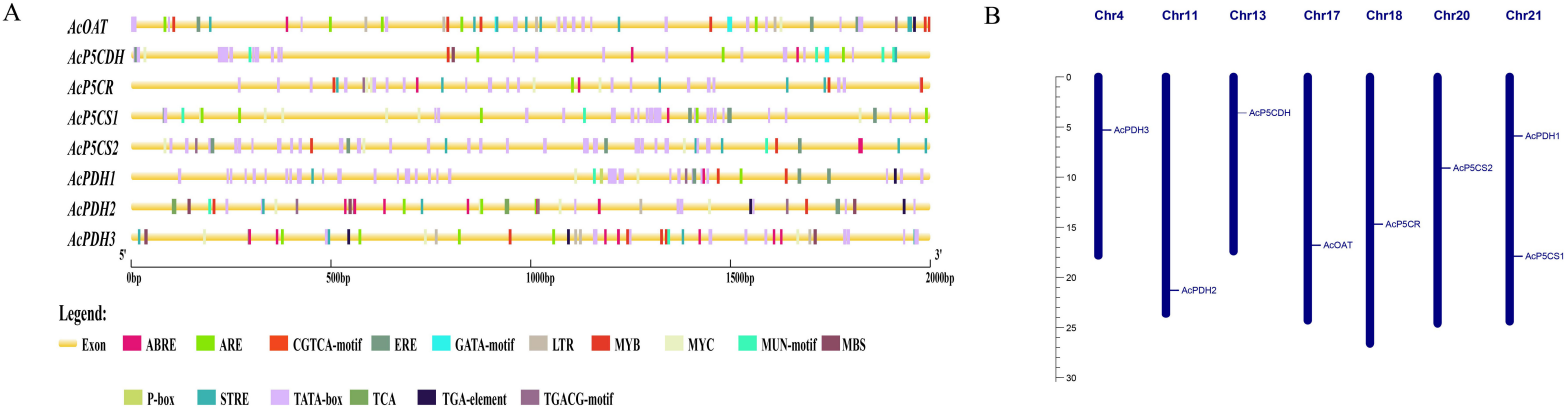
Cis-elements in promoter sequences and chromosomal distribution of the proline-metabolism gene family. **(A)** Predicted cis-regulatory elements within the 2000 bp upstream promoter regions of proline-metabolism genes, visualized as colored rectangles. **(B)** Genomic distribution of proline-metabolism genes across chromosomes, with chromosome numbers indicated on each ideogram.

Using the genomic data of *A.* c*hinensis*, an analysis of the 2000 bp upstream sequences of the eight genes involved in proline metabolism pathways revealed the presence of typical core promoter elements, including the TATA box, confirming their transcriptional regulatory function. All eight genes contain regulatory motifs responsive to Me-JA, specifically the CGTCA and TGACG elements (**Figure 2**). The promoter of all genes contains an abscisic acid-responsive cis-acting element (ABRE). The promoter of *AcP5CS1, AcP5CS2, AcOAT, AcPDH1*, and *AcPDH2* includes motifs associated with ethylene, namely ERE (**Figure 2**). The promoter of *AcPDH1* features ABRE and P-box elements responsive to abscisic acid and gibberellin. *AcPDH2*’s promoter contains ERE and TCA elements responsive to ethylene and salicylic acid. Beyond the regulatory elements, each gene’s promoter includes at least one stress-responsive cis-acting element (ARE, STRE, and TATA-box) (**Figure 2A**). The loci of eight genes associated with proline metabolism pathways were identified on multiple chromosomes, as shown in **Figure 2B**. Notably, Chromosome 21 harbored two genes, while the other chromosomes each hosted one gene.

### 2.3 Phylogenetic distribution of proline-metabolism gene family

**Figure 3:**
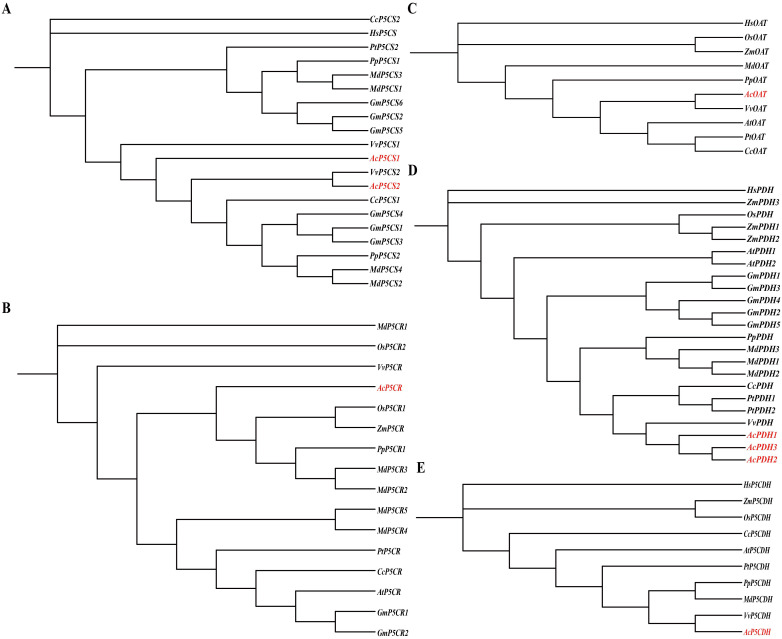
Phylogenetic trees of kiwifruit gene families—*P5CS* **(A)**, *P5CR* **(B)**, *OAT* **(C)**, *PDH* **(D)**, and *P5CDH* **(E)**—were reconstructed across diverse plant species. Sequence names are annotated with gene identifiers and species abbreviations, including model organisms such as *Populus trichocarpa* (Pt), *Oryza sativa* (Os), *Zea mays* (Zm), *Citrus clementina* (Cc), *Vitis vinifera* (Vv), *Glycine max* (Gm), *Prunus persica* (Pp), and *Malus domestica* (Md). Notably, the six genes originating from grapevines were highlighted in red font.

**Figure 3** illustrates the phylogenetic relationships of the *AcP5CS* (A), *AcP5CR* (B), *AcOAT* (C), *AcPDH* (D), and *AcP5CDH* (E) gene families, constructed using sequences from diverse plant species. These include model organisms such as *Arabidopsis thaliana* and *Populus trichocarpa*, as well as crops like *Oryza sativa*, *Zea mays*, *Citrus clementina*, *Vitis vinifera*, *Glycine max*, *Prunus persica*, and *Malus domestica*. The analysis indicates that *AcP5CS1* and *AcP5CS2* exhibit the closest phylogenetic relationships with *Vitis vinifera* (**Figure 3A**). Regarding the *AcP5CR* gene, kiwifruit clusters with sequences from *Oryza sativa*, *Zea mays*, *Malus domestica*, and *Prunus persica* suggest a shared evolutionary lineage (**Figure 3B**). The *AcOAT* gene from the kiwifruit exhibits a close relationship with *Vitis vinifera* (**Figure 3C**). The *AcPDHs* cluster with *Citrus clementina*, *Populus trichocarpa*, *Malus domestica*, and *Prunus persica*, indicating a common evolutionary lineage (**Figure 3D**). Finally, the phylogenetic analysis of *AcP5CDH* clusters closely with sequences from *Vitis vinifera*, underscoring a significant genetic relationship among these species (**Figure 3E**). Overall, the phylogenetic analysis suggests that the proline metabolism genes in kiwifruit have a notably closer genetic association with *Vitis vinifera*, *Malus domestica*, and *Prunus persica*, illustrating the evolutionary connections among these plant species.

### 2.4 Analysis of the tissue-specific expressions associated with proline-metabolism gene family

**Figure 4:**
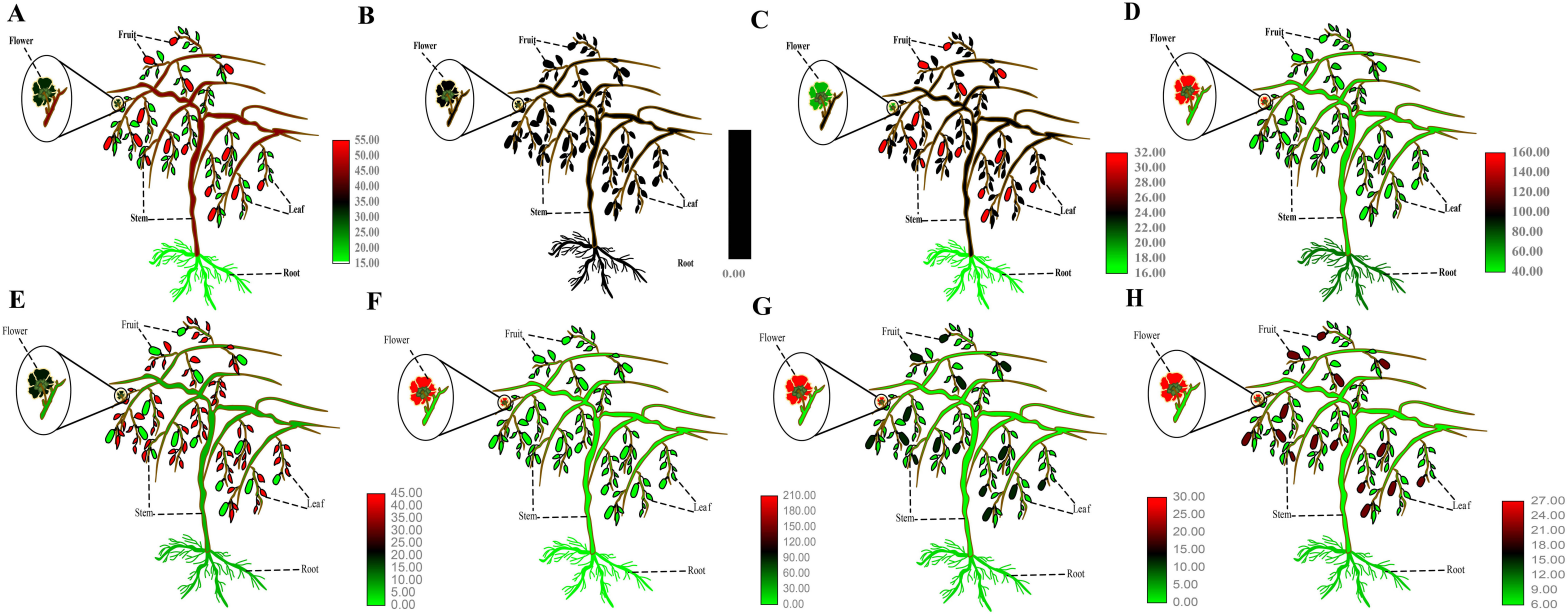
Electronic Fluorescent Pictograph (eFP) browser images of *AcP5CS1* **(A)**, *AcP5CS2* **(B)**, *AcP5CR* **(C)**, *AcOAT* **(D)**, *AcPDH1* ***(*E*)***, *AcPDH2* **(F)**, *AcPDH3* **(G)** and *AcP5CDH* **(H)** expression in different tissues.

The expression patterns of *AcP5CS1*, *AcP5CS2*, *AcP5CR*, *AcOAT*, *AcPDH1*, *AcPDH2*, *AcPDH3*, and *AcP5CDH* were analyzed in various kiwifruit tissues, such as root, stem, leaf, flower, and fruit. *AcP5CS1* gene expression was detected in multiple kiwifruit tissues, with the highest expression level observed in fruits under normal conditions (**Figure 4A**). *AcP5CS2* expression was relatively low across different tissues (**Figure 4B**). *AcP5CR* showed the highest expression level in fruits (**Figure 4C**). *AcOAT* displayed notably high expression in flowers (**Figure 4D**). *AcPDH1* exhibited higher expression levels in leaves (**Figure 4E**), *AcPDH2* exhibited relatively high expression levels across all tissues and the highest expression level in flowers (**Figure 4F**). *AcPDH3* and *AcP5CDH* exhibited the highest expression level in flowers (**Figures 4G, H**).

### 2.5 Expression patterns of proline-metabolism gene family under abiotic stresses and hormonal stress

**Figure 5:**
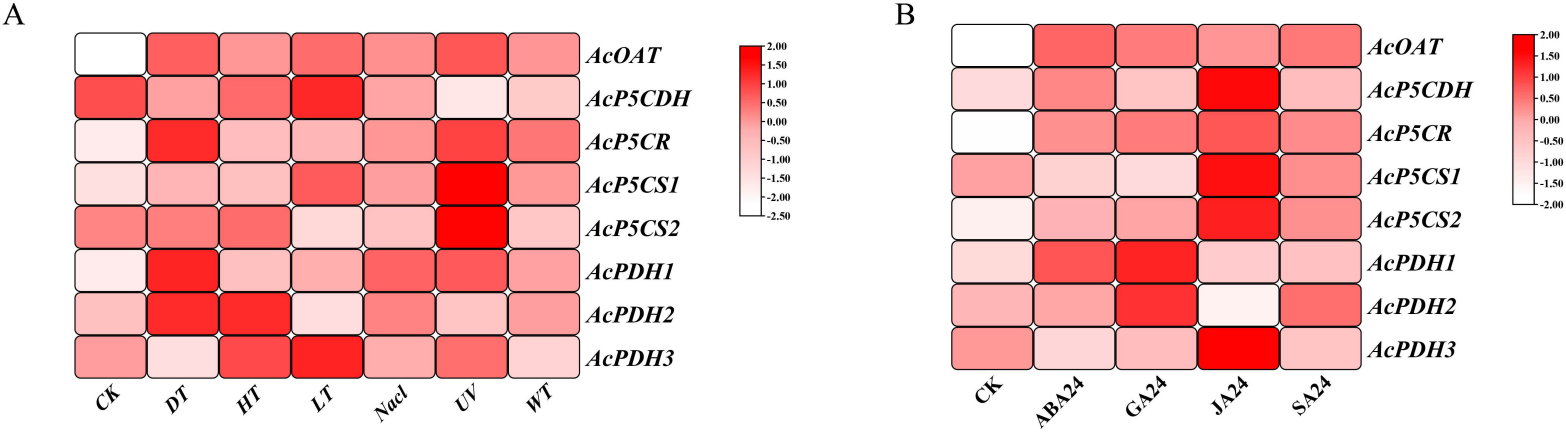
illustrates the expression profiles of the proline-metabolism gene family in response to abiotic stresses and hormone treatments. **(A)** Expression patterns of proline-metabolism gene family under abiotic stress conditions. **(B)** Expression patterns of proline-metabolism gene family following hormone treatments. The color bar adjacent to each figure indicates the log2 transformed FPKM (fragments per kilobase of exon per million reads mapped) values, representing normalized gene expression levels. Detailed explanations for all abbreviations (“CK” for control, “DT” for drought treatment, “HT” for heat temperature treatment, “LT” for low temperature treatment, “Nacl” for salt treatment, “UV” for ultraviolet radiation treatment, “WT” for waterlogging treatment, “ABA” for Abscisic Acid hormone treatment, “GA” for Gibberellic Acid hormone treatment, “JA” for Jasmonic Acid hormone treatment, “SA” for Salicylic Acid hormone treatment, “WT” for waterlogging treatment, “WT” for waterlogging treatment.) were used in the figure.

The proline-metabolism gene family exhibited significant upregulation under abiotic stresses (**Figure 5A**). Notably, *AcOAT*, *AcP5CR*, *AcP5CS1*, and *AcPDH1* genes displayed markedly higher expression levels following NaCl stress, while the *AcP5CDH* gene was downregulated under NaCl stress. *AcOAT*, *AcP5CR*, *AcPDH1*, and *AcPDH2* demonstrate strong induction. *AcP5CS2* was uniquely expressed during cold (LT) stress. In response to hormone treatments, all genes except for *AcPDH1/2* showed strong upregulation under JA treatment (**Figure 5B**). It’s interesting that *AcPDH1/2* showed strong upregulation under GA 211 treatment (**Figure 5B**). *AcOAT* was markedly upregulated under multiple hormonal treatments (**Figure 5B**). *AcOAT*, *AcP5CR*, *AcP5CS1/2* and *AcPDH1* exhibited significant induction in response to Abscissic acid (ABA) (**Figure 5B**). *AcOAT*, *AcP5CR*, *AcP5CS2* and *AcPDH2* exhibited a significant increase in expression in response to SA treatment (**Figure 5B**).

### 2.6 Verification of *AcOAT*, *AcP5CR*, *AcP5CS1* and *AcPDH1* genes expression under NaCl stress by qRT−PCR

**Figure 6:**
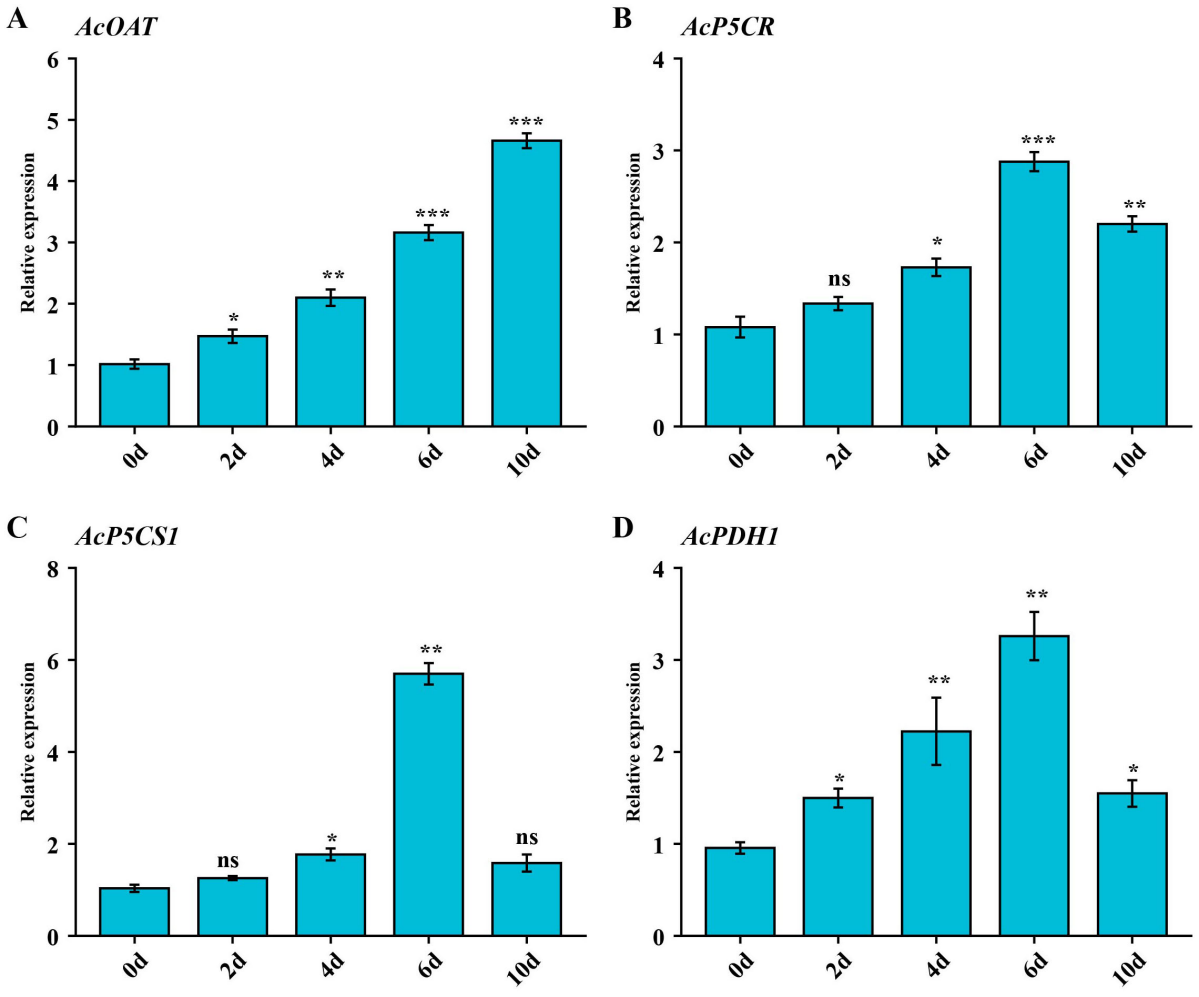
The expression levels of the proline-metabolism gene family genes at days 0, 2, 4, 6, 8 and 10 of salt stress, respectively. **(A)** Expression patterns of *AcOAT* gene under salt stress conditions. **(B)** Expression patterns of *AcP5CR* gene under salt stress conditions. **(C)** Expression patterns of *AcP5CS1* gene under salt stress conditions. **(D)** Expression patterns of *AcPDH1* gene under salt stress conditions. Error bars represent the mean ± standard deviation from three biological and technical replicates. Asterisks denote statistically significant upregulation or downregulation of gene expression in response to NaCl stress, as determined by Student’s t-test (p* < 0.05, *p* < 0.01, *p* * < 0.001). “ns” indicates no statistically significant difference.

We selected the *AcOAT*, *AcP5CR*, *AcP5CS1*, and *AcPDH1* genes for qRT-PCR analysis further to validate the expression pattern of genes under salt stress. Under salt treatment, the expression levels of *AcP5CR*, *AcP5CS1*, and *AcPDH1* in the leaf tissue were significantly induced, exhibiting similar expression trends and peaking on 6d (**Figures 6B, D**). The expression levels of *AcOAT* increased gradually during the salt treatment period (**Figure 6A**). These results indicate these genes’ potential role in alleviating salinity’s adverse effects.

### 2.7 The correlation between all transcription factors with the expression of *AcP5CS1* under salt stress and *AcNAC30* regulates *AcP5CS1* expression by directly activating and binding to its promoter

**Figure 7:**
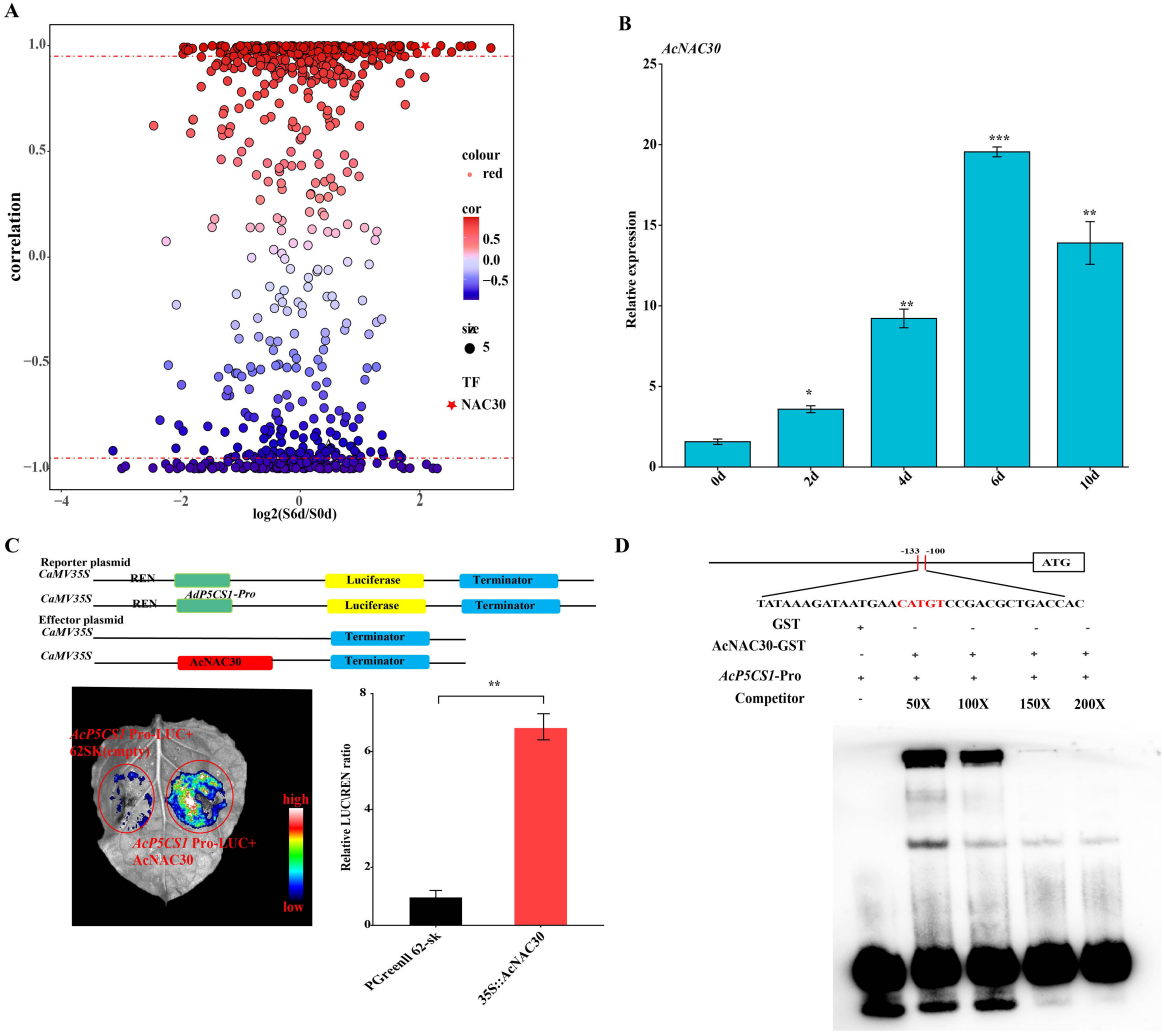
AcNAC30 Regulates directly targets and transcriptionally modulates the *AcP5CS1* promoter. **(A)** A correlation analysis of all transcription factors was conducted to evaluate their expression patterns relative to *AcP5CS1* based on RNA-seq results. The x-axis represents the log2 (FPKM ratio of salt treatment at 6 days to control at 0 days), while the y-axis denotes the correlation values between *AcP5CS1* and each transcription factor. Pentagons indicate the AcNAC30 transcription factor, which exhibited a strong correlation (R ≥ 0.95, red dashed lines) with the expression patterns of *AcP5CS1*. **(B)** The expression levels of *AcNAC30* genes under salt stress. **(C)** A dual-luciferase (LUC) assay in *Nicotiana benthamiana* leaves was conducted to assess the transcriptional activation of the *AcP5CS1* promoter by AcNAC30. The control group utilized an empty vector. Representative images depict the outcomes, and the LUC/Renilla luciferase (REN) activity ratio was quantified to confirm the AcNAC30-mediated activation of *AcP5CS1*. Error bars represent the standard deviation (SD). Statistical significance (*p < 0.05, *p* < 0.01, *p < 0.001) was determined using Student’s *t*-test. **(D)** Electrophoretic mobility shift assay (EMSA) demonstrated that *AcNAC30* binds specifically to defined regions of the *AcP5CS1* promoter.

The RNA-seq data of kiwifruit subjected to salt treatments were analyzed. The correlation results indicated a strong association between the *AcNAC30* transcription factors (TFs) and the expression of *AcP5CS1* (R ≈ 0.99; **Figure 7A**). The expression levels of *AcNAC30* were significantly induced, peaking at 6 days under salt treatment. A similar expression trend was also observed for *AcP5CS1* (**Figure 7B**).

To elucidate the transcriptional regulation of *AcP5CS1* by *AcNAC30*, dual-luciferase reporter assays were conducted in tobacco protoplasts. Compared to the control group injected with an empty vector, the co-expression of *AcNAC30* and *AcP5CS1*::LUC exhibited stronger relative fluorescence intensity and significantly increased enzyme activity (**Figure 7C**). These results suggest that *AcNAC30* positively regulates the expression of the target genes *AcP5CS1*. (**Figure 7C**). To further validate the direct interaction between AcNAC30 and the *AcP5CS1* promoter, an electrophoretic mobility shift assay (EMSA) was performed. The results revealed that AcNAC30 binds explicitly to the HSE motif in the *AcP5CS1* promoter, as demonstrated by a dose-dependent reduction in the electrophoretic mobility shift with increasing concentrations of unlabeled cold probe (0×–200×) (**Figure 7D**). These findings demonstrate that AcNAC30 directly regulates *AcP5CS1* expression by binding to the motif (CATGT) within its promoter region.

### 2.8 Salt tolerance of transgenic Arabidopsis overexpressing *AcP5CS1*

**Figure 8:**
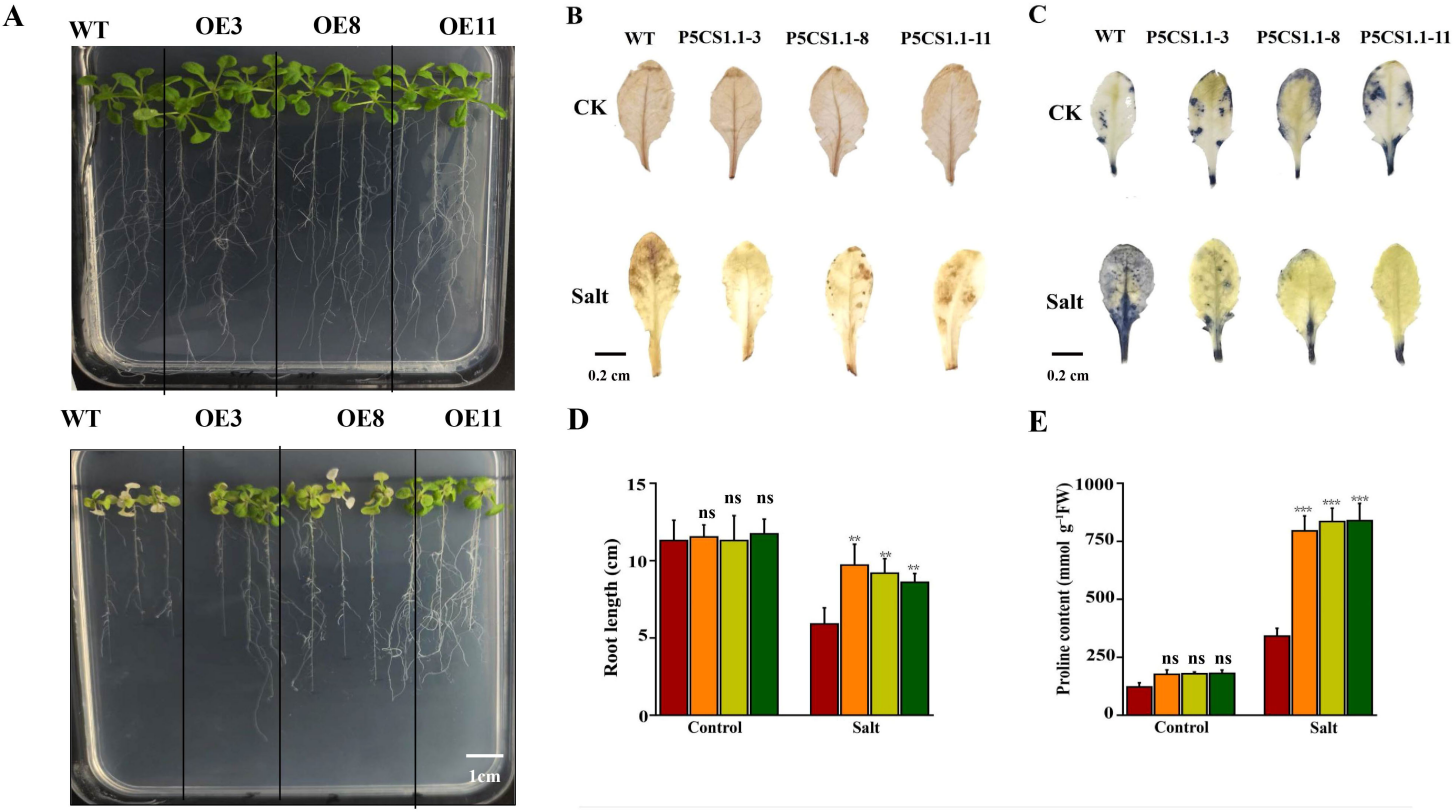
Overexpression of *AcP5CS1* enhances salt tolerance. **(A)** Phenotypic observations and root length measurements under varying NaCl concentrations. **(B)** DAB histochemical staining of Arabidopsis subjected to salt treatment. **(C)** NBT histochemical staining of Arabidopsis subjected to salt treatment. **(D)** Root length comparisons of wild-type (WT) and *AcP5CS1*-transgenic Arabidopsis seedlings under different NaCl concentrations. **(E)** Under varying NaCl concentrations, there was proline content in wild-type (WT) and AcP5CS1-transgenic Arabidopsis seedlings. Error bars represent averages from three biological and technical replicates. Asterisks indicate significant upregulation or downregulation of the corresponding gene under different treatments as determined by t-test (*p* < 0.01, *p < 0.001).

To elucidate the functional characterization of *AcP5CS1* under salt stress, three lines of transgenic Arabidopsis, designated as OE3, OE8, and OE11, were developed in this study. Under normal conditions (referred to as control), the transgenic plants exhibited robust growth, showing no noticeable differences. Following salt treatment, the OE lines displayed significantly more extensive and heavier leaves than wild-type plants (**Figure 8A**). The accumulation of H_2_O_2_ and O_2_^−^ in WT and OE Arabidopsis was investigated under salt stress using DAB and NBT staining. The results indicated a significant decrease in staining intensity in the branches of OE plants compared to WT plants under salt stress conditions (**Figures 8B, C**). This suggests that *AcP5CS1* plays a positive role in reducing the accumulation of H_2_O_2_ and O_2_^−^. The growth parameters—root length and proline content of seedlings overexpressing the *AcP5CS1* gene were considerably more significant than those of the wild-type plants (**Figures 8D, E**). These results suggest that the overexpression of *AcP5CS1* enhances tolerance to salt stress.

## 3 Discussion

Kiwifruit, a relatively new and recently domesticated fruit crop, enjoys considerable consumer popularity due to its exceptionally high vitamin C content (Han et al., 2023; Liu et al., 2023; Wang et al., 2024). Meanwhile, the yield and quality of kiwifruit are significantly impacted by various biotic and abiotic stresses. Abiotic factors such as salinity, temperature extremes, drought, and waterlogging adversely affect plant growth and development. Proline, an amino acid, benefits plants exposed to diverse stress conditions. In addition to its role as a superior osmolyte, proline performs three essential functions during stress: it acts as a metal chelator, an antioxidative defense molecule, and a signaling molecule (Ghosh et al., 2022). Stressful environments induce an overproduction of proline in plants, subsequently aiding stress tolerance (Alvarez et al., 2022). This is achieved by maintaining cellular turgor or osmotic balance, stabilizing membranes to prevent electrolyte leakage, and regulating reactive oxygen species (ROS) concentrations to avert plant oxidative bursts. Under high salt stress, proline (PRO) levels in kiwifruit leaves showed an increasing trend compared to the control treatment (Zhong et al., 2019; Abid et al., 2020). Given the significance of proline’s functions, genes related to proline metabolism have been investigated across multiple species (El Moukhtari et al., 2020) but not yet in kiwifruit. In the current study, eight proline metabolizing genes have been identified in kiwifruit (**Table 1**). Kiwifruit possesses two *AcP5CSs*, one *AcP5CR*, one *AcOAT*, three *AcPDHs*, and one *AcP5CDH*, consistent with earlier research in Arabidopsis, rice (Arabia et al., 2021) and rose (Adamipour et al., 2020). Our research demonstrated that many constituents within a homogenous group portray a comparable exon/intron framework, echoing the patterns detected in alfalfa (**Figure 1**) (Min et al., 2023). The genes *AcP5CS1*, *AcP5CS2*, *AcOAT*, *AcP5CDH*, and *AcP5CR* demonstrate the most potent phylogenetic associations with *Vitis vinifera*, reaffirming the conclusions from earlier research (**Figure 2**) (Yue et al., 2023). These findings suggest the proline metabolism-related gene family has remained markedly conservative throughout evolutionary processes.

The expression of proline biosynthesis and degradation genes is closely associated with various stress conditions, including salinity, drought, dehydration, submergence, heat, and cold (Kaur and Asthir, 2015). Proline biosynthesis in most plant species is predominantly driven by a two-step enzymatic process involving the enzymes P5CS and P5CR, employing glutamate as a substrate. In our study, the expression levels of the *AcP5CS1* and *AcP5CR* genes in kiwifruit were significantly upregulated under salt, drought, and heat stress (**Figures 5**, **6**). Moreover, the expression levels of these genes were positively correlated with proline content, consistent with the findings reported in Arabidopsis, rice, and potato (Liu et al., 2019; Arabia et al., 2021). Guan et al. revealed that overexpressing *PvP5CS1* and *PvP5CS2* improved salt tolerance in switchgrass (Guan et al., 2020). All *PbP5CS* members exhibited differential regulation in response to biotic and abiotic stresses, suggesting that these genes likely mediate plant defense mechanisms in pear (Ma et al., 2022). In conditions of salt stress, transgenic Arabidopsis overexpressing *SaP5CS2* demonstrated a high level of tolerance, with an accumulation of increased proline levels and a simultaneous reduction in H_2_O_2_ content (Zhao et al., 2022). Comparable findings were reported in this study. Liu et al. reported that the expression levels of *P5CS2* and *P5CR* in sweet sorghum leaves increased significantly following 3 hours of treatment with 20% PEG (Cui et al., 2024). The overexpression of *P5CR* resulted in increased proline accumulation in soybeans, thereby enhancing drought tolerance (Sharma and Dietz, 2006). These results suggest that *P5CSs* and *P5CRs* may facilitate the conversion of glutamate to proline. Conversely, in the catabolic pathway, proline can be converted to glutamate through the sequential actions of proline dehydrogenase (ProDH) and pyrroline-5-carboxylate dehydrogenase (P5CDH) (Arabia et al., 2021). Proline dehydrogenase (PDH), also known as proline oxidase (ProDH), demonstrated a significant increase in gene expression in response to salt, drought, and cold stress (**Figure 5**), corroborating the findings reported by Freitas et al (de Freitas et al., 2019). However, it has also been reported that proline degradation is inhibited during abiotic stress, as PDH transcription is activated by rehydration but repressed by dehydration (Hayat et al., 2012). In grapevines, the expression patterns of these genes under abiotic stresses indicated that *P5CS1*, *OAT*, and *PDH* could be significantly induced by treatments with salt, dehydration, polyethylene glycol (PEG), and hydrogen peroxide (H_2_O_2_). Furthermore, the ornithine pathway is capable of synthesizing proline, beginning with the transamination of ornithine by ornithine delta-aminotransferase (OAT), resulting in the production of GSA and P5C, which are subsequently converted to proline (Iqbal et al., 2014). We observed that the expression levels of the ornithine aminotransferase (OAT) gene were significantly upregulated following salt, drought, cold, heat and UV treatment (**Figures 5**, **6**). Plant hormones regulate plant growth and development, as well as stress responses, and are crucial in numerous physiological and biochemical processes (Verma et al., 2016). In this study, we analyzed the expression profiles of the proline metabolism-related genes under various hormone treatments using transcriptome data. Approximately 62% of the proline metabolism-related genes exhibited a significant increase in expression in response to SA treatment (**Figure 5B**). La et al. documented that SA pretreatment mitigated drought-induced superoxide radical (O2 -) accumulation, resulted in increased proline accumulation along with enhanced expression of proline synthesis-related genes (*P5CS1*, *P5CS2*, and *P5CR*) in *Brassica napus* (Yan et al., 2021). The expression of *AcPDH1*, *AcP5CS2* and *AcP5CR* significantly increased in response to ABA treatment. These findings are consistent with those reported by Sharma et al (Sharma and Verslues, 2010).

Transcription factors (TFs) are pivotal regulators of plant responses to salt stress. Among these, NAC proteins—comprising no apical meristem (NAM), *Arabidopsis thaliana* transcription activation factors (ATAF1/2), and cup-shaped cotyledon (CUC2)—represent one of the largest plant-specific TF families (Singh et al., 2013). These proteins regulate diverse biological processes, including plant development, responses to biotic and abiotic stresses, and hormone signaling pathways. Wang et al. (2021) reported that a member of the NAC transcription factor family, *StNAC053* (Soltu.DM.06G017300), responded to salt stress in potato. In *StNAC053* transgenic Arabidopsis plants, the *P5CS* gene exhibited marked upregulation relative to the control group under salt stress (Wang et al., 2021). Additionally, overexpression of *PcNAC25* in potato plants resulted in heightened levels of *P5CS1* expression and enhanced its salt resistance (Wu and Wang, 2025). The correlation analysis revealed a robust correlation between AcNAC30 (Actinidia09980) transcription factors (TFs) and the expression of *AcP5CS1*, with a correlation coefficient of R ≈ 0.99 (**Figure 7A**), suggesting that AcNAC30 protein likely interacts with the promoter of the *AcP5CS1* gene to boost plant salt resistance. Li et al. demonstrated that *AvNAC030* is likely to modulate the expression of AtP5CS1, a gene involved in proline synthesis induced during salt stress, potentially enhancing plant salt tolerance (Li et al., 2021). *StNAC015310* might promote proline accumulation under salt stress by increasing the expression levels of proline metabolic pathway genes (Jing et al., 2022). Therefore, we speculated that *AcNAC30* likely enhances proline accumulation under salt stress by upregulating the expression of proline metabolic pathway genes. Considering the results of LUC and EMSA, we conclude that the transcription factor *AcNAC30* may activate *AcP5CS1* expression by directly binding its promoter (**Figures 7C, D**). Consequently, the findings of this study suggest that salt stress promoted the glutamate pathway and concurrently activated the ornithine pathway, resulting in proline accumulation, which in turn enhances plant abiotic stress tolerance.

## 4 Materials and methods

### 4.1 Plant material

The “Hongyang” kiwifruit variety, aged 5 years, was obtained from the College of Horticulture at Anhui Agriculture University and served as the principal material in this study. Various tissues were collected, including young leaves (specifically the fourth to fifth leaves from the shoot apices when the newly growing shoots reached approximately 60 cm in length), stems (located at the apices of newly growing shoots with a diameter of approximately 2 mm), flower, root, and fruits (around 6 cm in diameter).

### 4.2 Identification and sequence analysis of proline metabolism genes in kiwifruit

The seven genes (AT2G39800, AT3G55610, AT5G14800, AT5G46180, AT3G30775, AT5G38710, and AT5G62530) known to encode proline metabolism enzymes in Arabidopsis were utilized as query sequences in a search of the kiwifruit genome database (http://kiwifruitgenome.org/) (Hare et al., 1999; Yue et al., 2023). The online ProtParam tool (https://web.expasy.org/protparam/) was employed to analyze the molecular weight (MW) and theoretical isoelectric points (pI) of the proteins. Furthermore, the identified candidate genes were verified using the Blast tool in NCBI (https://blast.ncbi.nlm.nih.gov/Blast.cgi) based on their homology with the corresponding genes in Arabidopsis.

### 4.3 Analysis of gene structure, promoter analysis and phylogenetic tree

The gene structures of *P5CSs*, *P5CRs*, *OATs*, *PDHs*, and *P5CDHs* genes were visualized using the online Gene Structure Display Service (Liu et al., 2019) to illustrate the arrangement of exons and introns. The conserved motifs within these genes were identified using MEME Suite 5.1.1 (Guan et al., 2020). The gene promoters positioned 2000 bp upstream of the initiation codon (ATG) were extracted from the kiwifruit genome (http://kiwifruitgenome.org/). The putative cis-regulatory elements within the promoter sequences were predicted using the PLACE database (http://bioinformatics.psb.ugent.be/webtools/plantcare/html/) and presented with the TBtools software (https://www.tbtools.com/). The physical location map of chromosomes was visualized by MapInspect 1.0. Phylogenetic trees were constructed using MEGA-X software, applying the neighbor-joining (NJ) method with the specified parameters: Bootstrap method with 1000 replications (Kumar et al., 2018). The study encompassed related plant species, including model organisms and crops such as *Oryza sativa*, *Zea mays*, *Citrus clementina*, *Vitis vinifera*, *Glycine max*, *Prunus persica*, *Malus domestica*, *Arabidopsis thaliana*, and *Populus trichocarpa*.

### 4.4 Expression patterns analysis of proline metabolism genes in different tissues, hormone treatments, and in response to abiotic stresses

The sample preparation procedure involved the treatment of kiwifruit with abiotic stresses, following the methodology employed in a prior study (Ling et al., 2023). Tissue culture seedlings of *A. chinensis* ‘Hongyang’ variety (HY) were transplanted into a perlite and sand soil mix (3:1, v/v) and cultivated in a growth chamber under controlled conditions: 18°C (night), 24°C (day), 60–80% relative humidity, and a 14/10 h photoperiod (06:00–20:00). Irrigation was provided every other day. After two months, the seedlings were randomly allocated to six groups for stress treatments. Heat and cold stresses were induced by placing the seedlings in chambers set to 48°C and 4°C, respectively, with harvesting at 6 h post-treatment. Salt, drought, and waterlogging stresses were applied by immersing the seedlings in 0.6% NaCl for 6 days, flooding for 7 days, and drying for 14 days. Untreated seedlings served as the control (CK). The paragraph was refined to match academic writing standards by improving spelling, grammar, clarity, conciseness, and overall readability. The RNA-seq data of kiwifruit generated are publicly available in the National Center for Biotechnology Information (NCBI) repository under the accession number PRJNA1028382. Proline metabolism gene expressions in different tissues, hormone treatments, and in response to abiotic stresses were assessed using quantitative real-time PCR (qRT-PCR) on the Biorad CFX96 real-time PCR system with the ChamQ SYBR qPCR Master Mix (Vazyme, China). Total RNA isolation was performed as previously described. The relative gene expression levels were calculated using the 2^−ΔΔCt^ method. Primers for these genes were designed using Primer 3 software (http://frodo.wi.mit.edu/, accessed on 30 April 2023) (**Supplementary Table S1**). A bar chart was generated using R software (geom_bar function in the ggplot2 library). The eFP heat maps of different tissues were created using Tbtools software (Chen et al., 2020).

### 4.5 Dual-luciferase reporter assay and electrophoretic mobility shift assay

The full-length coding sequence (CDS) of *AcNAC30* was cloned into the pGreenII 62-SK vector to generate the effector construct, while the promoter region of *AcP5CS1* was inserted into the pGreenII0800-LUC vector to create the reporter construct. Both constructs were transformed into *Agrobacterium tumefaciens* strain EHA105 (primers listed in **Supplementary Table S2**). The bacterial suspension (10 mM MgCl_2_, 10 mM MES, 150 µM acetosyringone, pH 5.6) was grown to an OD_600_ of 0.6 and co-infiltrated into 4-week-old tobacco (*Nicotiana benthamiana*) leaves. After 2–3 days of incubation at 23°C, control leaves were sprayed with water, while experimental leaves were treated with 1000 mg/kg ethephon. Luciferase activity was measured using a dual-luciferase assay kit (YEASEN, Shanghai, China). Leaf discs were harvested, and luminescence signals for firefly luciferase (LUC) and Renilla luciferase (REN) were quantified using a microplate luminometer (Berthold Centro LB960). Promoter activity was expressed as the LUC/REN ratio. Fluorescence in tobacco leaves was visualized and photographed using a chemiluminescence imager (Tanon 5200, Shanghai, China).

The open reading frame (ORF) of *AcNAC30* was incorporated into the pGEX-4T-1 vector, designed to express a GST-tagged recombinant protein. This modified vector was then introduced to E. coli BL21 (DE3) cells (TransGen, Beijing, China) (primers listed in **Supplementary Table S2**). Upon achieving an OD600 of 0.6 at 37°C, the expression of the GST- AcNAC30 fusion protein was induced using 0.5 mM IPTG. The conditions for protein expression were fine-tuned to a 10–12 hour period at 28°C to ensure the proper folding and solubility of the GST-tagged protein. The fusion protein underwent purification using a GST magnetic bead purification kit (Beaver, Suzhou, Jiangsu, China), with the procedure adhering to the manufacturer’s guidelines. A DNA probe comprising binding sequences from the *AcP5CS1* promoter (TATAAAGATAATGAACATGTCCGACGCTGACCAC) was formulated through PlantCare (http://bioinformatics.psb.ugent.be/webtools/plantcare/html/, accessed on 15 April 2023) and synthesized by Tsingke Biotechnology (Nanjing, Jiangsu, China). This probe was tagged with biotin via the EMSA Probe Biotinylation Kit (Beyotime Biotechnology, Haimen, Jiangsu, China). The DNA gel mobility shift assay used the Chemiluminescent EMSA Kit (Beyotime Biotechnology, Jiangsu, China). For this procedure, the GST- AcNAC30 protein extract was mixed with the biotinylated probe in a binding buffer and incubated at room temperature for 30 minutes. The resulting mixture was loaded onto a 6% denaturing polyacrylamide gel and subjected to electrophoresis. Post-separation, the gel was developed using a chemiluminescent substrate (Beyotime Biotechnology), and the DNA-protein complexes were captured using a Kodak Image Station (Rochester, NY).

### 4.6 Phenotypic analysis of *AcP5CS1*-overexpressing *t*ransgenic Arabidopsis under salt treatment

The open reading frame (ORF) of *AcP5CS1* was cloned into the pCAMBIA1305 vector using primers listed in **Supplementary Table S2**. The confirmed recombinant construct (*AcP5CS1-pCAMBIA1305*) was subsequently introduced into *Arabidopsis thaliana* ecotype Columbia (Col-0) plants via the floral dip method, employing *Agrobacterium tumefaciens*-mediated transformation. After sowing the T3 generation positive homozygous transgenic Arabidopsis thaliana seeds on 1/2MS solid medium containing hygromycin as a selection marker, the seedlings were grown for 10 days. Healthy transgenic and wild-type seedlings with consistent root length and size were transferred to MS medium containing different concentrations of NaCl (0 mmol/L and 150 mmol/L), with three seedlings of each genotype planted on each plate. After 10 days of salt treatment, the phenotypes of transgenic Arabidopsis were observed, photographed, and sampled. The root length of both wild-type and transgenic Arabidopsis was determined using Image J 1.8.0. The wild-type and transgenic Arabidopsis leaves were immediately removed from the culture medium, rapidly frozen in liquid nitrogen, and stored at -80 °C in an ultra-low temperature freezer for future experimental needs. The proline determination procedure was conducted following the acid-ninhydrin method with minor modifications, as described by Shabnam et al. (2016). Initially, 0.1 g of the samples were placed into 2 mL of a 3% sulfosalicylic acid solution and subjected to extraction by boiling in a water bath for 15 minutes. Subsequently, 1 mL of the supernatant, 1 mL of glacial acetic acid, and 2 mL of acid-ninhydrin was prepared and further boiled in a water bath for 60 minutes. After allowing it to cool, 1 mL of toluene was added to extract the resulting red product. The upper red toluene layer was then utilized to conduct spectrophotometry at a wavelength of 520 nm. The proline content was determined by referencing an L-proline standard curve, and the experiment was performed in triplicate using three biological replicates. The leaves of transgenic and control Arabidopsis plants subjected to salt treatment were stained using tissue-specific chemical staining for ROS detection with 3,3’-diaminobenzidine (DAB) and nitroblue tetrazolium (NBT). The pre-treated and post-treated leaves were immersed in DAB or NBT staining solution and subjected to vacuum infiltration for 20 minutes. Subsequently, the leaves were incubated at room temperature in darkness for 8 hours, followed by decolorization with 95%-100% (v/v) ethanol until the leaves turned white. The decolorized leaves were stored in water and photographed.

## 5 Conclusions

In this study, we conducted a comprehensive analysis of proline metabolism-related genes in kiwifruit, employing a genome-wide approach to identify eight specific genes: *AcP5CS1*, *AcP5CS2*, *AcP5CR*, *AcOAT*, *AcPDH1*, *AcPDH2*, *AcPDH3*, and *AcP5CDH*. We found that the *AcP5CS1* gene has highlighted its potential role in mediating salt tolerance, suggesting that it may be essential for the plant’s adaptive responses to saline conditions. We utilized ectopic expression techniques to evaluate how *AcP5CS1* influences salt stress tolerance in Arabidopsis, providing empirical evidence of its functional significance. Furthermore, the results from dual-luciferase assays and electrophoretic mobility shift assays confirmed that *AcNAC30* can effectively bind to the promoter element of the *AcP5CS1* gene, indicating a regulatory mechanism. Collectively, our findings establish a solid foundation for future investigations into the roles of *AcP5CS1* genes in enhancing kiwifruit resilience to various abiotic stresses. By elucidating the mechanisms underlying *AcP5CS1* gene functionality, this research could significantly contribute to developing stress-tolerant kiwifruit varieties, ultimately benefiting agricultural practices and sustainability.

## Data Availability

The datasets presented in this study can be found in online repositories. The names of the repository/repositories and accession number(s) can be found in the article/**Supplementary Material**.
